# Magnetic spin–orbit interaction of light

**DOI:** 10.1038/s41377-018-0018-9

**Published:** 2018-06-27

**Authors:** Mengjia Wang, Hongyi Zhang, Tatiana Kovalevich, Roland Salut, Myun-Sik Kim, Miguel Angel Suarez, Maria-Pilar Bernal, Hans-Peter Herzig, Huihui Lu, Thierry Grosjean

**Affiliations:** 1 FEMTO-ST Institute, Université Bourgogne Franche-Comté, UMR CNRS 6174 15B Av. des Montboucons, 25030 Besancon Cedex, France; 2 Optics and Photonics Technology Laboratory, Ecole Polytechnique Fédérale de Lausanne (EPFL), Rue de la Maladière 71b, Neuchâtel, CH-2000 Switzerland; 3 Department of Optoelectronic Engineering, Guangdong Provincial Key Laboratory of Optical Fiber Sensing and Communications, Jinan University, Guangzhou, 510632 China

## Abstract

We study the directional excitation of optical surface waves controlled by the magnetic field of light. We theoretically predict that a spinning magnetic dipole develops a tunable unidirectional coupling of light to transverse electric (TE) polarized Bloch surface waves (BSWs). Experimentally, we show that the helicity of light projected onto a subwavelength groove milled into the top layer of a 1D photonic crystal (PC) controls the power distribution between two TE-polarized BSWs excited on both sides of the groove. Such a phenomenon is shown to be solely mediated by the helicity of the magnetic optical field, thus revealing a magnetic spin-orbit interaction of light. Remarkably, this magnetic optical effect is clearly observed via a near-field coupler governed by an electric dipole moment: it is of the same order of magnitude as the electric optical effects involved in the coupling. This opens up new degrees of freedom for the manipulation of light and offers desirable and novel opportunities for the development of integrated optical functionalities.

## Surprising new effects of the magnetic properties of light

The magnetic field carried by light can unexpectedly steer waves in structured media through optical spin-orbit interaction, according to researchers from France, Switzerland, and China. Until recently, it was thought that the magnetic field of light played very little role in the interactions between light and matter. Now, a team including Thierry Grosjean at Université Bourgogne Franche-Comté have used computer simulations and experiments to show that the magnetic component of a polarized light can control the directionality of Bloch surface waves on a one-dimensional photonic crystal (a dielectric multilayer). The phenomenon implies for the first time that light undergoes ‘a spin-orbit interaction’ that solely involves its magnetic spin density. The findings could open new opportunities for the manipulation of light in opto-electronic devices and the local probing of the magnetic spin density.

## Introduction

The magnetic field of light is often considered to be a negligible contributor to the light–matter interaction. However, with the advent of left-handed metamaterials^1–4^, nanophotonics has recently been used to investigate the magnetic response in nanostructures to reveal the hidden magnetic part of the light–matter interaction, e.g., to achieve negative refractive indices^4^, control magnetic transitions in matter^5–7^, map optical magnetic fields^8–13^, and study magnetic effects at optical frequencies^14–16^. In this study, we show that the magnetic field of light also has the desirable ability to control light coupling into optical surface waves. 

Optical angular momenta are manifestations of the polarization and spatial degrees of freedom of light^17^. Remarkably, spin and orbital momenta are not independent quantities: spin angular momentum (SAM) can be converted into orbital angular momentum and vice versa^18^. Such a spin–orbit interaction (SOI) has recently drawn much interest for applications involving light manipulation^19–25^. For example, the SOI has demonstrated the remarkable property of controlling the propagation direction of guided modes, such as surface plasmon, fiber, and waveguide modes, leading to the concept of spin-controlled unidirectional waveguiding^19,26–35^. Robust spin-controlled unidirectional waveguiding relies on the transverse SAM arising in evanescent waves^36–39^. With the use of a subwavelength (dipolar) coupler, the longitudinal SAM of an impinging wave can be transferred into the transverse SAM of the evanescent tail of a guided mode, leading to spin-directional coupling for the guided mode^19,28,29^. So far, such investigations have mainly focused on the rotating electric component of light as the source of the SAM for originating the transverse spin-direction coupling^29,36,37^ (the spin density of light is usually described by rotating electric and magnetic optical fields^38,40,41^).

**Fig. 1:**
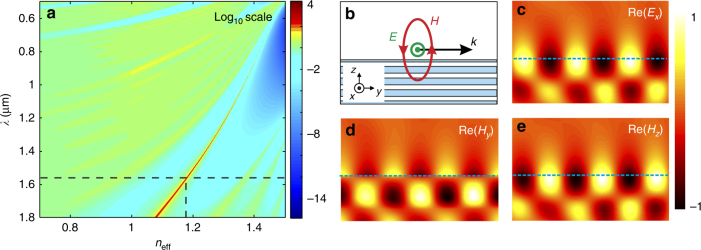
**Theoretical description of a TE-polarized Bloch surface wave. a** Dispersion diagram of the 1D photonic crystal with a log-scale color bar. The structure generates a photonic bandgap at the middle of which the dispersion curve of a BSW is observed. **b** Schematic diagram of the electromagnetic field distribution for a TE-polarized BSW. **c** Simulation result for the real part of the electric field component *E*_*x*_ parallel to the sample surface. **d**, **e** Simulation results for the real part of *H*_*y*_ and *H*_*z*_, respectively

Here we introduce the concept of spin-direction locking mediated solely by a rotating magnetic light field. We study the light coupling in Bloch surface waves (BSWs) by projecting circularly polarized light onto a subwavelength scatterer that is used as a near-field coupler. BSWs are surface modes on top of a one-dimensional (1D) photonic crystal (PC)^42–46^. Importantly, when the BSW is TE-polarized, its evanescent tail in the surrounding medium is described by a rotating magnetic field (Fig. 1b) instead of a rotating electric field as for transverse magnetic (TM) polarized surface plasmons or the guided mode of a nanofiber^18,37^. We numerically show that the tunable unidirectional excitation of TE-polarized BSWs can be realized using a spinning magnetic dipole (MD) source^34,47^, demonstrating that the rotating magnetic field of a BSW carries SAM. Using a subwavelength groove as a light-to-BSW converter, we observe that the directionality of the incoupled light is helicity dependent. From the intrinsic spin properties of TE-polarized evanescent waves and an analytical model for the coupling, we infer that the resulting spin-controlled directional coupling is mediated by the magnetic optical field, thus revealing a magnetic SOI for light. Despite the electric dipole (ED) nature of the subwavelength groove, this magnetic optical effect is found to be of the order of magnitude of the electric effects involved in the coupling process. It is noteworthy that such a magnetic SOI is intrinsic to optics; it is not related to the SOI in matter controlled by a static magnetic field^48^.

## Materials and methods

### Numerical models

All the numerical simulations were carried out using the finite difference time domain method (FDTD, commercial code).

The two-dimensional (2D) FDTD model used for the calculations of the dipole-to-BSW near-field coupling consists of an area about the dipole spanning ± 9 μm along the *y* direction and ± 5.5 μm along the *z* direction. The system is invariant along the *x* direction. The 1D PC design is described below. All four boundaries for the computation volume are terminated with perfectly matched layers to avoid parasitic unphysical reflections around the structure. The grid resolution is 10 nm. For the MD, two simulations are realized with the dipole moment oriented along the *y* and *z* axis. In both cases, the electromagnetic fields across the structure are recorded. Then, BSW excitation with a spinning MD is reconstructed from these two sets of simulations (see the Supplementary Fig. S1). In both cases, the helicity of the dipole moments are defined analytically to match the helicity of the magnetic field for the BSWs under study.

The three-dimensional (3D) FDTD model used for the calculation of the light/BSW coupling with a single groove consists of a volume that spans ± 5 µm along both the *x* and *y* directions perpendicular and parallel to the groove, respectively. The groove, with both a width and a depth of 600 nm, is engraved into the top layer of the 1D PC considered in this study. It is located at *x* = *z* = 0 and extends in the *y* direction across the computation volume. The simulation spans 4.9 µm below the 1D PC top surface in the glass substrate and terminates 2 µm in air, beyond the multilayer. The operating wavelength is 1.55 µm. All six boundaries for the computation volume are terminated with perfectly matched layers to avoid parasitic unphysical reflections around the structure. The non-uniform grid resolution varies from 20 nm for portions at the periphery of the simulation to 8 nm within and near the top layer. To excite the BSWs, a Gaussian beam is launched onto the groove. Its propagation axis is tilted by 80° with respect to the surface normal. Due to computation limitations, a beam waist of 1.5 µm is chosen to limit the computation volume. Two simulations are conducted using incident beams with TE and TM polarizations with respect to the surface. In both cases, the intensities of the excited BSWs on the right and left sides of the groove are recorded. Afterward, the various BSW excitation scenarios are reconstructed from these two simulations. To this end, the various input polarizations are expressed in the TE/TM local coordinate frame of the incidence beam.

### Experimental illumination system

The illumination system consists of two lenses coupled to a polarizer and a quarter-wave plate (QWP), (see details in Supplementary Material and Supplementary Fig. S2). The first lens (*f* *=* 33 mm) collimates the light emerging from a laser source coupled into a single mode fiber, whereas the second lens (*f* *=* 50 mm), positioned closer to the sample, focuses the collimated beam onto the groove. The polarizer and QWP are positioned between the two lenses. The polarizer ensures a TM linearly polarized incident beam with respect to the sample surface. It is followed by the QWP for manipulating the polarization ellipticity of the incident beam. By rotating the QWP, the polarization of the incident beam is tuned from linear to circular polarization, with intermediate elliptic polarization states. Importantly, in this configuration, the orientation of the polarization ellipse varies with the orientation of the QWP. A linear combination of the BSW intensities is then carried out with the projection amplitudes used as the coefficients for the linear combination.

## Results and discussions

BSWs are generated by a 1D PC consisting of a stack of six pairs of silicon dioxide and silicon nitride layers, with refractive indices of 1.45 and 1.79 (at *λ* *=* 1.55 µm), and thicknesses of 492 nm and 263 nm, respectively. The multilayer lies on a glass substrate (refractive index of 1.5) and is covered with a thin 80 nm-thick layer of silicon nitride. Figure 1a shows the dispersion diagram of the structure (calculated using the impedance approach^49^). This diagram shows a photonic bandgap, which contains the dispersion curve for a BSW. Given the 1D PC design, this surface mode is TE-polarized, as shown in Fig. 1b-e. We observe that *Hy* (Fig. 1d) is shifted by a quarter wavelength with respect to *Hz* (Fig. 1e) along the propagation direction *y* of the surface wave, thus revealing the helicity of the optical magnetic field along the transverse *x* direction, as shown in Fig. 1b. The transverse SAM for the surface wave is thus carried solely by its rotating magnetic field; the electric field shows no helicity (see Fig. 1c).

**Fig. 2:**
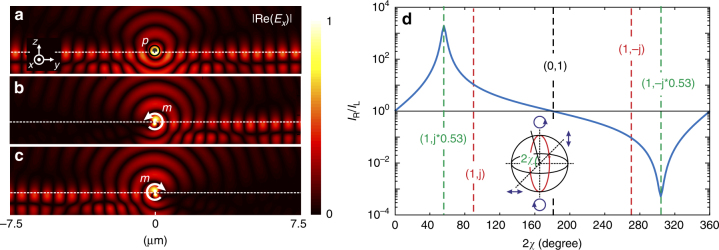
TE-polarized Bloch surface waves excited with ED and MD sources. Simulation by FDTD of the coupling of **a** an ED source oriented along the *x* axis and **b**, **c** a spinning MD source, to a TE-polarized BSW. All three results show, in false colors, the absolute value of the real part of the electric field ($\left| {Re\left( {E_x} \right)} \right|$). The MD rotates either **b** anti-clockwise or **c** clockwise. **d** Directionality factor (ratio of the electric intensities for the left and right BSWs) for various MD polarizations. The MD polarization ellipticity is changed along the path shown in red in the Poincare sphere (see inset). The MD moment is also expressed at specific values of the ellipticity angle 2*χ* related to the Poincare sphere

We numerically study the coupling of single ED and MD to a TE-polarized BSW. To this end, the dipoles are considered to be positioned 10 nm above the top surface of the 1D PC described above, which radiate light in a continuous wave regime at *λ* *=* 1.55 µm (details in Materials and Methods section). The ED is oriented along the *x* direction along the surface, i.e., parallel to the electric field of the TE-polarized BSW. The MD rotates in the (*yz*) plane perpendicular to the surface, i.e., in the helicity plane of the rotating magnetic field of the TE-polarized BSW. Figure 2 shows a snapshot of the resulting electric field amplitude along the (*yz*) plane, for the ED (Fig. 2a) and MD (Fig. 2b, c) excitations. The MD, whose dipole moment is $\vec m \propto \vec e_y \pm j \ast 0.53\vec e_z$ ($j = \sqrt { - 1}$), rotates either anti-clockwise (Fig. 2b) or clockwise (Fig. 2c). In these figures, the field distributions around the dipoles are saturated to provide a better view of the light distributions at the structure surface. The simulations are carried out using the 2D FDTD method. With the ED, the BSW is symmetrically excited on both sides of the point-like source. No directionality is observed in the optical coupling process. In contrast, the optical coupling process becomes unidirectional for the spinning MD. Figure 2d shows the ratio of the electric intensities for the left and right BSWs, for various MD polarizations. The MD polarization is tuned along the path shown in red in the Poincare sphere (see figure inset). In this case, only the ellipticity angle (2*χ*) for the MD moment varies. Our directionality factor (intensity ratio) becomes larger than 10^3^ at 2*χ* = 55.8° and smaller than 10^−3^ at 2*χ* = 304.2°. Therefore, at these two particular angles, the portion of the incoupled power that propagates in one of the two possible directions becomes larger than 99.9% of the total incoupled power. These results reveal a tunable unidirectional optical coupling controlled by the magnetic field of light. They also confirm that the rotating magnetic field of a TE-polarized BSW carries SAM.

From an experimental point-of-view, it is possible to realize a tunable directional coupling for BSW with a spinning MD using a dielectric sphere showing magnetic resonances^6,50,51^ directly deposited on top of the 1D PC. This bead can then be illuminated with a circularly polarized beam at near-grazing incidence, following the electric spin-controlled excitation process of surface plasmons^29^. We will show here that magnetic directional coupling can be clearly demonstrated even with a standard subwavelength groove directly engraved on top of the 1D PC.

**Fig. 3:**
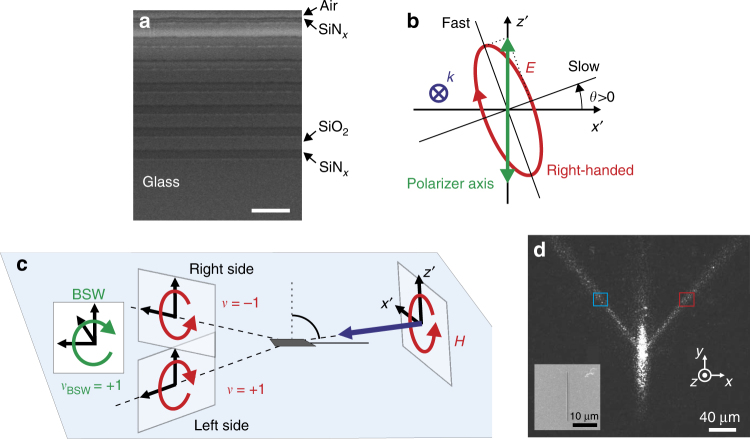
**A simple all-dielectric platform unveiling the magnetic spin-orbit interaction. a** SEM cross-section of the 1D photonic crystal (scale bar: 1 µm). **b** Schematic diagram of the elliptic polarization (electric optical field) generated by the polarizer (fixed) and the quarter-wave plate (rotating). **c** Schematic diagram of the magnetic field handedness in the helicity planes of the right and left BSWs for an incident right-handed polarization. The handedness is defined here by the parameter *v*: we have *v* = + 1 and *v* = − 1 for the clockwise and anticlockwise rotating magnetic fields, respectively. **d** Far-field optical image of the BSW obtained via excitation at *λ* = 1.55 µm of a 600 nm wide and deep groove. The groove is 20 μm long. The laser beam is incident from air onto the top surface of the 1D PC at almost the grazing angle (incidence angle: 80°, see Supplementary Fig. S2). This image originates from light scattering at the sample top surface. The incident light is linearly (TM) polarized here, to reveal the two symmetric BSW propagation directions provided by the phase-matching condition. Figure inset: SEM top view of the groove milled into the top surface of the 1D photonic crystal

To fabricate the 1D PC described above, thin layers of silicon oxide and silicon nitride are deposited alternately by plasma-enhanced chemical vapor deposition onto a glass wafer. A cross-section of the multilayer realized by a focused ion beam (FIB) reveals the design detailed above (Fig. 3a). Then, the sample is covered by a 100 nm-thick chromium layer and a 600 nm wide and deep groove is milled by FIB over a length of 20 µm. Finally, the chromium is removed. The inset of Fig. 3d shows a scanning electron microscope (SEM) image for the resulting structure.

The structure is characterized in the far-field by projecting a slightly focused beam of controlled polarization onto the sub-wavelength groove at an incidence angle of approximately 80°. The structure is imaged in reflection mode with an objective (× 20, numerical aperture = 0.4) coupled to an infrared camera (see details in the Supplementary Material and Supplementary Fig. S2). Due to light scattering at the free surface of the 1D PC, a direct real-time mapping is possible for the surface waves excited on both sides of the groove. Figure 3d shows the far-field images for the surface around the groove under illumination. The incident beam is TM linearly polarized with respect to the sample surface, leading to a symmetric scattering pattern. It is noteworthy that the definition of the TE/TM polarizations for the incident light and the BSWs are related to different local coordinate frames, and thus they should not be directly compared. In the context of our study, a TM-polarized incident wave can excite a TE-polarized BSW. The bright elongated spot along the *y* axis is the cross-section of the excitation beam along the surface. The two narrow rays on both sides of the excitation spot are traces of the BSWs excited by the subwavelength groove. Linear momentum conservation imposes a tilt angle for the BSW propagation direction with respect to the groove direction (*y*) that is predicted to be approximately 33.8°; it is measured to be approximately 36°.

We then study the distribution of the incoupled power between the two surface waves as a function of the incident polarization. The polarization is defined by the angle *θ* between the fast axis of the QWP and the polarizer. When = *k* 90° *k*=0,1,2,3, the polarization is linear whereas a circular polarization is realized for *θ* = 45° + *k*90°, *k* = 0,1,2,3. For intermediate angles, the polarization is elliptical. On the one hand, the incident polarization is defined by a rotating QWP and a fixed polarizer. In that case, the polarization ellipse for the emerging light (that is incident onto the 1D PC) rotates with the crystalline axes of the QWP (see Fig. 3b). Such a polarization property leads to an incident light field whose electric and magnetic amplitudes have, by projection, a 2*θ* dependence. The energy coupling to the right and left BSWs thus undergoes a 4*θ* dependence as it is homogeneous to the intensity (this point will be discussed in detail later). As our near-field coupler (the groove, i.e., dielectric scatterer) is mainly driven by an ED moment (per unit length), the energy coupling to the BSWs can be assumed to be mediated by the electric optical field. The energy coupling is, therefore, helicity independent since the electric field of a TE-polarized BSW is linearly polarized (Fig. 1b). On the other hand, the handedness of the light waves leaving the quarter wave plate shows a 180° periodicity with respect to the angle *θ*. Such a property originates from the universal 180° periodicity for the helicity dependent optical phenomena. As only the magnetic field of the TE-polarized BSW is rotating (Fig. 1b), any helicity dependent contribution to the excitation process for the surface wave will arise from a magnetic optical effect. Importantly, the incident magnetic field shows opposite handedness when projected onto the helicity planes of the right and left BSWs (see Fig. 3c). For instance, a right-handed incident polarization leads to a magnetic field rotating clockwise (*v* = + 1) and anticlockwise (*v* = − 1) in the helicity planes of the left and right BSWs, respectively (Fig. 3c). Such a configuration is favorable for spin-controlled unidirectional excitation of the BSWs, with the magnetic field of the BSW being described by the handedness *v*_BSW_ = + 1. A right-handed (left-handed, respectively) incident polarization would thus direct TE-polarized BSWs to the left side (to the right side, respectively) of the groove. Such a spin-controlled contribution to the coupling process leads to a 2*θ-*dependent power distribution between the two BSWs excited on both sides of the groove.

**Fig. 4:**
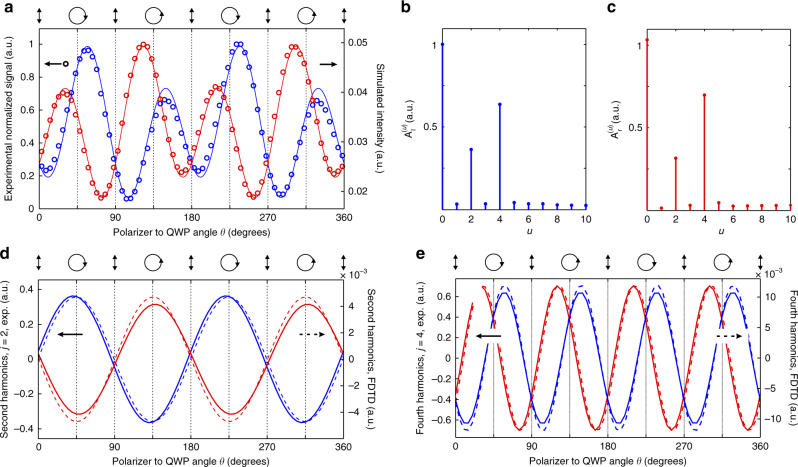
**Magnetic spin-orbit interaction steers Bloch surface waves. a** Detected signals (circles) and simulated intensities (FDTD method, solid lines) on the right and left BSWs as a function of the angle *θ* between the quarter-wave plate and the polarizer. The curves related to the right and left BSWs are represented by red and blue colors, respectively. **b**, **c** Spectrum (amplitude) of the experimental blue and red curves of **a**, obtained by Fourier transformation. Coefficient *u* defines the harmonic orders for the Fourier series. **d**, **e** Representation in the real space of the non-null harmonics for the two Fourier series shown in **b** and **c**: **d** second harmonics and **e** fourth harmonics. Experimental and numerical curves are shown by the solid and dashed lines, respectively

We experimentally acquired images of the structure while varying the polarization of the incident beam. For each image recorded at a specific polarization state, we integrate the signal detected over two square areas located symmetrically with respect to the groove (shown in light red and blue colors in Fig. 3d). Finally, the resulting values *S*_*r*_ and *S*_*l*_ measured on the right and left BSWs, respectively, are plotted as a function of the angle *θ* (Fig. 4a). The experimental plots are represented by solid lines together with the simulation results obtained with the 3D FDTD method (see details in the Materials and Methods section).

Figure 4a shows *S*_*r*_ and *S*_*l*_ as a function of *θ S*_*r*_ and *S*_*l*_ are described by sinusoidal functions, shifted by ~ 30° from each other, with the amplitudes modulated by a sinusoidal function. The experimental results and simulation predictions are in good agreement. As expected, the coupling process for light in the BSWs is asymmetric except for the linear (TM) incident polarization (*θ*=*k* 90° *k*=0,1,2,3). In this case, the optical system is fully symmetric with respect to the groove and the two curves for *S*_r_ and *S*_*l*_ merge. As discussed above, the electric and magnetic contributions to the BSW excitation process show a 4*θ* and 2*θ* dependence, respectively. Therefore, a Fourier analysis for *S*_*r*_ and *S*_*l*_ may assist identification of these electric and magnetic optical effects. By Fourier transforming these two functions (cf. Figure 4b, c), we see that they can be simply expressed analytically as:

1 $$S_i\left( \theta \right) = A_i^{(0)} + A_i^{\left( 2 \right)}\sin (2\theta + \phi _i^{\left( 2 \right)}) + A_i^{\left( 4 \right)}\sin (4\theta + \phi _i^{\left( 4 \right)})$$

where *i*=*r,l*, and the coefficients $A_i^{(u)}$ and $\phi _i^{(u)}$(*u* = 0, 2, 4) are constant. Coefficients $A_i^{(u)}$ and $\phi _i^{(u)}$ (*u* = 2, 4) are given by the Fourier transform of *S*_*r*_ and *S*_*l*_.

We see that in Fig. 4d the second harmonic components relative to the left and right BSWs are almost in opposition, i.e., shifted by 180°. The fourth harmonics (cf. Figure 4e) undergo a shift of approximately 30° initially evidenced in Fig. 4a. Importantly, the local maxima and minima of the second harmonic component closely coincide with the right and left-handed circular polarization states. Moreover, changing incident polarization handedness inverts the distribution for the incoupled light in the right and left BSWs. The second harmonic contribution to the BSW coupling is, therefore, helicity dependent. In contrast, the fourth harmonics of the Fourier series stay unchanged when the input polarization handedness is reversed (See Fig. 4e). Therefore, the fourth harmonic contribution to the BSW coupling is independent of the helicity of the light.

As subwavelength scatterers are optically governed by an ED moment, one may consider that a subwavelength groove on top of a 1D PC interacts with the electric field of an incoming wave to transfer energy to the BSWs. Following this pure electric model, and assuming an incident plane wave, the previously defined coefficients *S*_*r*_ and *S*_*l*_ become proportional to the coupling rates:

2 $$R_i = \alpha \left| {\vec e_i \cdot \vec E_{inc}(\vec r_0)} \right|^2$$

where *i* *=* *r*, *l* denotes the right and left sides of the groove and *α* is a constant. $\vec E_{inc}(\vec r_0)$ is the incident electric field at a single point for the coordinate $\vec r_0$ along the subwavelength groove. $\vec e_i$ is the unit vector in the direction of the electric field of the emerging right and left BSWs. When plotted as a function of the angle *θ*, *R*_*r*_, and *R*_*l*_ are described by two sinusoids showing a 4*θ* dependence and shifted by 8*°* (see Supplementary Material and Supplementary Fig. S4). Moreover, changing the polarization handedness does not interchange the values for the two coefficients, which indicates that pure electric coupling of the incident light to the TE-polarized surface waves is helicity independent. By comparing Supplementary Fig. S4 and Fig. 4e, we see that *R*_*r*_ and *R*_*l*_ closely match the fourth harmonic function of Eq. 1. The unbalanced electric coupling is due to the asymmetric projections for the electric field onto $\vec e_r$ and $\vec e_l$. The larger angular shift observed between the couple of experimental curves (30° vs. 10° with our model) may be because the scattering properties of our 600 nm large (i.e., *λ*/2.5) groove-like coupler slightly deviates from the dipole emission.

The modulation of the electric coupling by a helicity dependent optical process (Fig. 4) is not predicted by our analytical model. As noted above, only the magnetic field of the TE-polarized BSW is rotating, with the electric field showing zero helicity. A helicity dependent process for such waves, thus, solely involves the magnetic field of the light. We plotted, as a function of *θ*, the ellipticity factor for the magnetic field (plane-wave illumination) projected onto the helicity planes of the right and left BSWs (i.e., the planes perpendicular to the transverse spin momentum of the surface waves). Details of the calculation are given in the Supplementary Material. The ellipticity curves show a periodicity of 2*θ* and opposite values when the polarization handedness is changed (see the Supplementary Fig. S5). These curves closely resemble the second harmonic functions of Eq. 1 (Fig. 4d). Therefore, the second harmonic contribution to the optical coupling is solely controlled by the magnetic field of the light.

Remarkably, the magnetic effect is clearly visible using a dielectric scatterer described by an ED moment (per unit length). Figure 4b, c show that its contribution is larger than 45% of the electric contribution to the coupling. Despite the extremely low response of the scatterer to the magnetic field of the impinging wave, the rotating magnetic field incident at an electric scatterer provides the initial conditions to direct a large portion of the incoupled energy to the right or to the left BSW depending on the polarization handedness. The second harmonic curves shown in Fig. 4d thus describe a magnetic spin-directional coupling, as shown in Fig. 2. In the experimental case, however, the phase matching between the incident light and the BSW is mediated by the electric optical field given the ED nature of the scatterer. The rotating incident magnetic field incident at the groove, which is less affected by the scatterer, controls the directionality of the launched surface waves. This explains the 4*θ* dependence of the experimental coupling process (Fig. 4) that is comparatively not observed for MD excitation: Fig. 2c shows a directionality curve with only a 180° periodicity. The BSW excitation undergoes light-to-BSW electric field projection rules that accompany the phase matching. A subwavelength resonant particle or antenna, whose resonance is described by a MD moment, would cancel this electric component of the coupling. Such a configuration is, however, beyond the scope of this paper.

## Conclusion

We described a new magnetic effect in a light-matter interaction called the magnetic SOI of light. On the basis of this magnetic SOI, we showed that an elliptically polarized MD develops a tunable unidirectional coupling of light into TE-polarized BSWs: depending on the helicity of the MD, the surface waves propagate upstream or downstream. The underlying phenomenon is a transverse spin-direction coupling in BSWs, but with the spin momentum here solely described by a rotating magnetic light field. This phenomenon was demonstrated using a simple subwavelength groove used as a light-to-BSW converter. Despite the ED nature of this coupler, the magnetic effect is of the same order of magnitude as the electric effects in the coupling process. In this particular case, the magnetic field is not involved in the light-to-BSW phase matching process, which is responsible for the transfer of energy to the BSW, but instead, controls the directionality of the incoupled energy. By using couplers that can develop magnetic resonances^6,50,51^, a pure magnetic tunable unidirectional coupling is experimentally possible, in accordance with our theoretical predictions. In addition to the fundamental questions raised regarding magnetic optical control of light-matter interaction, our results open possibilities for controlling optical flows in ultra-compact architectures. The results can also be generalized to all TE-polarized guided modes. Reciprocally, BSWs can also be used as probes to locally investigate the magnetic polarization properties of light. Compared to the (plasmonic) nanoprobes that are currently used, which are limited to measuring the electric spin density only, such BSW probes would enable measurement of the optical magnetic spin density^38,40^.

## Electronic supplementary material

Supplementary material
