# Supplementary material for: Magnetic spin–orbit interaction of light
Source: Light Sci Appl. 2018 Jun 27;7:24. doi: 10.1038/s41377-018-0018-9 (PMC6107028; doi:10.1038/s41377-018-0018-9)
Supplement: Supplementary file 1 — Supplementary material [file 41377_2018_18_MOESM1_ESM.docx]

**Magnetic spin-orbit interaction of light**

Mengjia Wang^1^, Hongyi Zhang^1^, Tatiana Kovalevich^1^, Roland Salut^1^, Myun-Sik Kim^2^, Miguel Angel Suarez^1^, Maria-Pilar Bernal^1^, Hans-Peter Herzig^2^, Huihui Lu^3,§^, and Thierry Grosjean^1,^*

^1^ FEMTO-ST Institute, Université Bourgogne Franche-Comté, UMR CNRS 6174 15B Av. des Montboucons, 25030 Besancon cedex, France

^2^ Optics & Photonics Technology Laboratory, Ecole Polytechnique Fédérale de Lausanne (EPFL), Rue de la Maladière 71b, Neuchâtel, CH-2000, Switzerland

^3^ Guangdong Provincial Key Laboratory of Optical Fiber Sensing and Communications, Department of Optoelectronic Engineering, Jinan University, Guangzhou 510632, China

^§^ email: [thuihuilu@jnu.edu.cn](mailto:thuihuilu@jnu.edu.cn)

* E-mail: [thierry.grosjean@univ-fcomte.fr](mailto:thierry.grosjean@univ-fcomte.fr),

Phone: +33 (0)3 81 66 64 17. Fax: +33 (0)3 81 66 64 23

**Supplementary Information**

Outline:

1. Simulation of the coupling of a spinning magnetic dipole to TE-polarized BSWs
2. Experimental setup
3. Analytical model of a pure electric coupling of light-to-BSW
4. Calculation of the ellipticity of an incident plane wave in the helicity plane of the TE-polarized BSWs

1. **
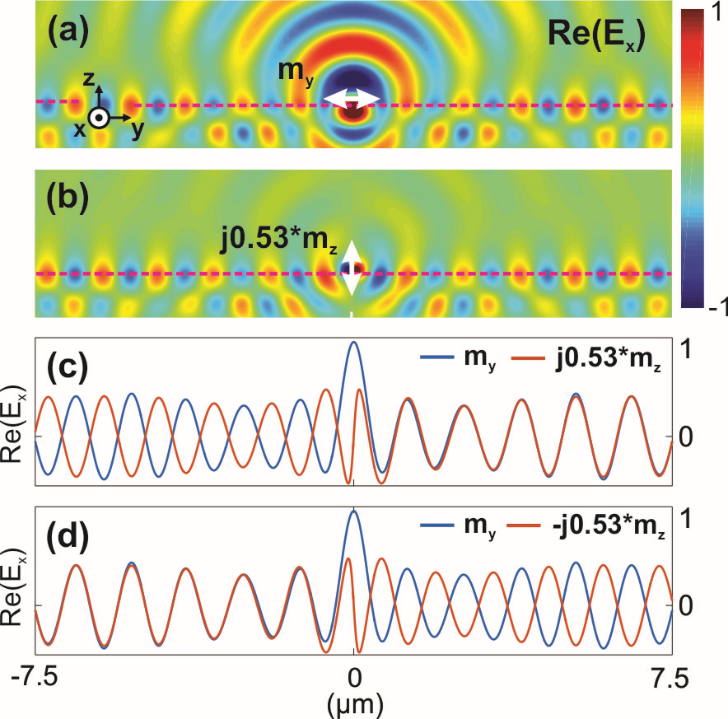
Simulation of the coupling of a spinning magnetic dipole to TE-polarized BSWs**

**Fig. S1.** (a) and (b) Snapshots (FDTD simulations) of the E_x_ component of the optical electric field produced in the (yz) plane by two MDs oriented along y and z axis, respectively. The MDs are positioned 10 nm away from the top surface of a 1D photonic crystal sustaining TE-polarized BSWs (top surface: (xy) plane). In these figures, the field around the dipoles is saturated in order to provide a better view of the light distributions across the structure. The field distribution in (b) is multiplied by $0.53j$. By adding/subtracting the results of (a) and (b), we achieve the field distributions across the 1D photonic crystal, produced by two spinning MDs of opposite handedness, which are described by dipole moments $\vec{m}$ proportional to $\vec{e_{y}}\pm0.53j \vec{e_{z}}$. (c) Electric field profiles ($Re(E_{x})$) along the dashed lines of (a) and (b), respectively. Adding these field profiles leads to the total electric field produced by a spinning MD moment proportional to $\vec{e_{y}}+0.53j \vec{e_{z}}$ (anti-clockwise rotation). (d) Electric field profiles with a spinning MD moment of $\vec{e_{y}}-0.53j \vec{e_{z}}$ (the field plotted in (b) is inverted, leading to a clockwise rotating MD). (c) and (d) show that the BSW coupling process is unidirectional and tunable with the helicity of light. For a spinning MD moment proportional to $\vec{e_{y}}+0.53j \vec{e_{z}}$ (see (c)), the BSWs generated by the two orthogonal MDs lead to destructive and constructive interference phenomena on the left and right sides of the MD, respectively. The opposite interference picture is observed for the MD moment proportional to $\vec{e_{y}}-0.53j \vec{e_{z}}$ (see (d)). Both configurations lead to a single propagation channel for the BSWs (on the right in (c) and on the left in (d)).

1. **Experimental setup**

The schematic diagram of the experimental setup is represented in Fig. S2. Light at λ=1.55 µm emerges from a tunable laser source (Agilent) coupled to a single mode fiber (SMF-28, Corning). It is collimated by the first lens ($f$ = 33 mm, Thorlabs) and focused by the second lens ($f$ = 50 mm, Thorlabs). The polarization of the collimated wave is manipulated by using a polarizer (LP, Thorlabs) and a quarter-wave plate (QWP, Thorlabs) positioned between the two lenses. The quarter-wave plate can be rotated at will with respect to the polarizer. Focused waves are projected directly on the groove at almost grazing incidence (incidence angle *β=80°*). The groove and its surrounding region covering the BSW propagation are imaged with an objective (20X, NA = 0.4) coupled to an infrared camera (GoldEye model G-033, Allied Vision Technologies GmbH). Far field imaging of the surface waves is rendered possible by the slight surface imperfection of the top layer of the 1D photonic crystal, which scatters the BSWs into the free space.


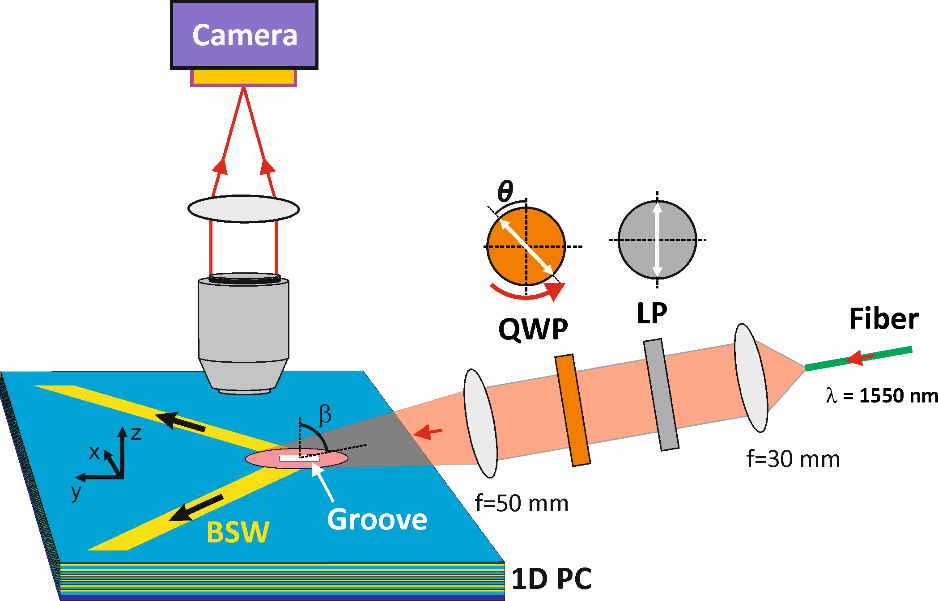


**Fig. S2.** Schematic diagram of the experimental setup. BSW: Bloch surface wave, PC: photonic crystal, QWP: quarter wave plate, LP: linear polarizer.

1. **Analytical model of a pure electric coupling of light-to-BSW**

Figure S3 illustrates the local coordinate frames $(x^{'},y^{'},z^{'})$ of an incident plane wave, and $(x^{L},y^{L},z^{L})$ and ${(x}^{R},y^{R},z^{R})$ of the BSWs propagating on the left and right sides of the groove, respectively. It also show the global coordinate frame $(x,y,z)$ linked to the surface of the 1D photonic crystal. The incidence angle of the plane wave is $\beta$.

The model of a pure electric coupling between the incident light and the BSW suggests the projection of the amplitude of the incident field to the unit vectors $\vec{e}=(1,0,0)$ expressed in the local coordinate frames $(x^{L},y^{L},z^{L})$ and ${(x}^{R},y^{R},z^{R})$. These vectors are represented in red in Fig. S3.


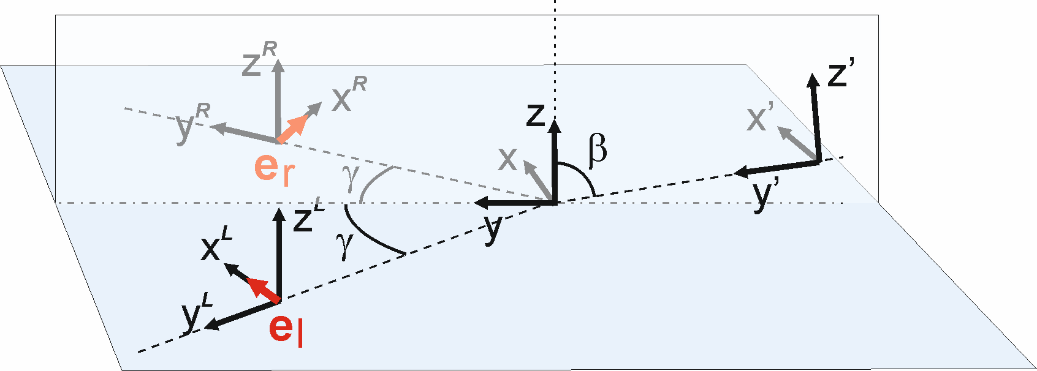


**Fig. S3.** Schematics of the field projection and corresponding global and local coordinate frames.

Assigning $\gamma$ to be the angle between the groove and BSW trajectories, $\vec{e}_{r}$ and $\vec{e}_{l}$ expressed in $\left( x,y,z \right)$ read:

$\vec{e}_{r}=\left[ \begin{matrix} \cos\gamma\\ -\sin\gamma\\ 0 \end{matrix} \right]$ (S1)

$\vec{e}_{l}=\left[ \begin{matrix} \cos\gamma\\ \sin\gamma\\ 0 \end{matrix} \right]$ (S2)

To define the incident electric field $\vec{E}_{inc}$, we assume that the incident plane wave, initially polarized along $z^{'}$ direction, is phase retarded by a quarter-wavelength along an axis tilted by an angle $\theta$ with respect to the polarizer axis ($y^{'}$-axis). We find, in the $\left( x,y,z \right)$ coordinate frame:

$\vec{E}_{inc}\propto\left[ \begin{matrix} -(1-j)\sin\theta\cos\theta\\ (\cos^{2} \theta+j\sin^{2} \theta)\cos\beta\\ (\cos^{2} \theta+j\sin^{2} \theta)\sin\beta\end{matrix} \right]$ (S3)

Following the coupling model proposed in the article, the coupling rates $R_{r}$ and $R_{l}$ of the incident wave to the right and left BSWs, respectively, read:

$R_{r}=\alpha\left| \left( 1-j \right)\sin\theta\cos\theta\cos\gamma+\left( \cos^{2} \theta+j\sin^{2} \theta\right)\cos\beta\sin\gamma\right|^{2}$, (S4)

$R_{l}={\alpha\left| \left( 1-j \right)\sin\theta\cos\theta\cos\gamma-(\cos^{2} \theta+j\sin^{2} \theta)\cos\beta\sin\gamma\right|}^{2}$, (S5)

where $\alpha$ is a constant. According to the phase matching condition (*i.e.*, linear momentum conservation) between the free space propagating incident wave and the BSWs, we have:

$\frac{\omega}{c}\sin\beta=k_{BSW}\cos\gamma$, (S6)


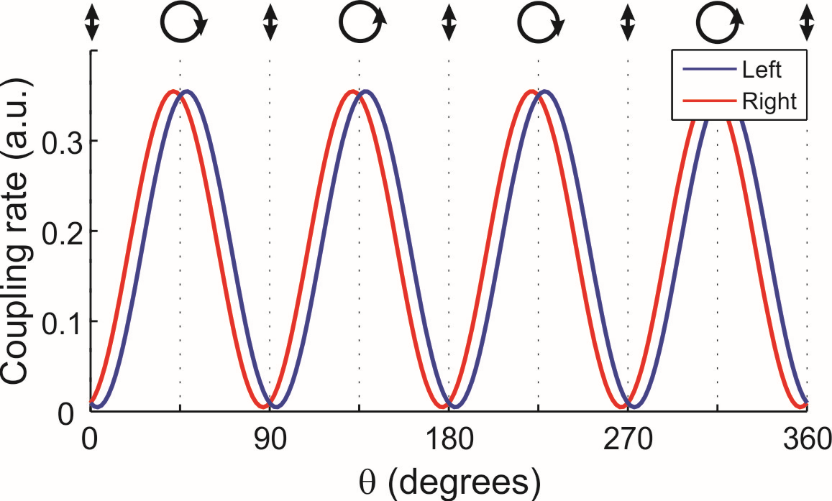
where $k_{BSW}$ is the wave vector of the BSWs. In our case, $n_{eff}=1.185$ and $\beta=80^{\circ}$, therefore we find $\gamma=33.8^{\circ}$.

**Fig. S4.** Plots of the coupling rates $R_{r}$ (red curve) and $R_{l}$ (blue curve) as a function of the angle $\theta$.

Fig. S4 shows $R_{r}$ and $R_{l}$ as a function of the angle $\theta$. The blue and red curves represent the coupling rates to the left and right BSWs. These two coefficients, which describe a pure electric coupling in the BSW excitation process, show a $4\theta$ dependence. They are also shifted by an angle of about 8°

1. **Ellipticity of the magnetic field of an incident plane wave in the helicity plane of the TE-polarized BSWs.**

According to the Maxwell-Faraday equation, the magnetic field of the incident plane wave can be expressed from Eq. S3 as:

$\vec{H}_{inc}=\frac{\omega\varepsilon}{k_{0}}\left[ \begin{matrix} \cos^{2} \theta+j\sin^{2} \theta\\ (1-j)\sin\theta\cos\theta\cos\beta\\ (1-j)\sin\theta\cos\theta\sin\beta\end{matrix} \right]$ (S7)

To examine the ellipticity of the incident magnetic field in the helicity planes of the BSW, one can make a transformation of $H_{inc}$ from the global coordinate frame $(x,y,z)$ to local ones ${(x}^{L},y^{L},z^{L})$ and ${(x}^{R},y^{R},z^{R})$. To this end, we define $\vec{H}_{i}=M_{i}\times\vec{H}_{inc}$, where $i=r,l$. $M_{r}$ and $M_{l}$ are the transformation matrices having the following forms for the right and left BSWs:

$M_{r}=\left( \begin{matrix} \cos\gamma& -sin \gamma& 0 \\ \sin\gamma& \cos\gamma& 0 \\ 0 & 0 & 1 \end{matrix} \right)$ (S81)

$M_{l}=\left( -\begin{matrix} \cos\gamma& \sin\gamma& 0 \\ \sin\gamma& \cos\gamma& 0 \\ 0 & 0 & 1 \end{matrix} \right)$ (S2)


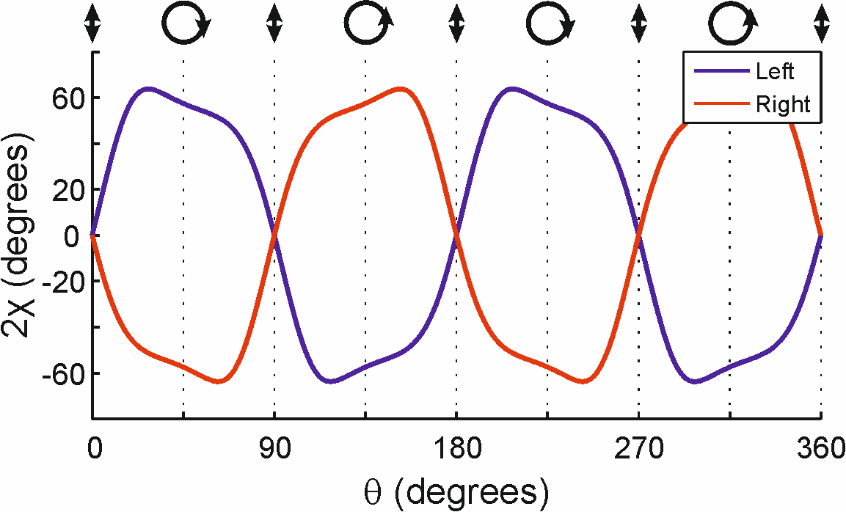
 The ellipticity of the incident magnetic field is defined by the polar angle ($2\chi$) of the Poincaré sphere.

**Fig. S5.** Ellipticity $2\chi$ of the incident magnetic field in the helicity planes ${(y}^{L},z^{L})$ and ${(y}^{R},z^{R})$ of the left and right TE-polarized BSWs.

Figure S5 represents $2\chi$ in the helicity planes ${(y}^{L},z^{L})$ and ${(y}^{R},z^{R})$ of the left and right BSWs, respectively, as a function of $\theta$. The two curves show an oscillating behavior with a $2\theta$ dependence and an amplitude of 63.7°. They are in opposition from each other, i.e., shifted by 180°. Switching polarization handedness changes the sign of the angle $2\chi$, it is obviously helicity-dependent.
